# Health-Related Quality of Life in Kidney Transplant Recipients

**DOI:** 10.5812/numonthly.9490

**Published:** 2013-05-30

**Authors:** Mariusz Niemczyk

**Affiliations:** 1 Department of Immunology, Transplant Medicine and Internal Diseases, Medical University of Warsaw, Poland

**Keywords:** Kidney Transplantation, Quality of Life

*Dear Editor,*

I have read with interest the paper of Tayyebi et al. (1) and I am impressed by the efforts that the authors made to elaborate the Iranian version of the kidney transplant questionnaire (KTQ-25) and I would like to congratulate the authors for the effect of their work. Renal transplantation is a method that improves both patient survival and quality of life, and is cost-effective in patients with end-stage renal disease compared to dialysis. Among these 3 factors, the quality of life is the most difficult to be estimated. Disease-specific tools designed for the assessment of health-related quality of life are of special value, since they are sensitive to slight, but important changes of the health status (2). Therefore, the impact of current advances in transplant technology and immunosuppression on patients' quality of life may be evaluated mainly with such tools, and this assessment is of importance in clinical decision-making.
